# Intermittent cold stress-induced experimental fibromyalgia model in mice - pharmacology and neurobiology

**DOI:** 10.1186/ar3607

**Published:** 2012-02-09

**Authors:** Kohei Araki, Michiko Nishiyori, Hiroshi Ueda

**Affiliations:** 1 Division of Molecular Pharmacology and Neurosciences, Nagasaki University Graduate School of Biomedical Sciences, Nagasaki 852-8521, Japan

## 

Stress-induced pain, as in Fibromyalgia (FM), is considered to be caused by intense events involving physical and psychological injury and is reinforced by successive stress. Previously, we have established a novel mice model of FM, using intermittent cold stress (ICS) exposure. Mice given ICS caused abnormal pain, including mechanical allodynia and hyperalgesia to nociceptive thermal and chemical stimuli, which lasted for more than 2 weeks. In contrast, those given constant cold stress (CCS) did not. The abnormal pain was generalized, female-predominant and specific for A-delta and A-beta, but not C-fiber-stimuli in the electrical stimulation-induced nociceptive test. The mechanical allodynia induced by ICS was effectively suppressed by intraperitoneal or intracerebroventricular injection of gabapentin. The potency and duration of anti-allodynia effects were much higher and longer, respectively, than the neuropathic pain induced by sciatic nerve injury. Taken together, these findings indicate that mice given ICS manifest most of characteristics observed in fibromyalgia patients in terms of pharmacology and pain physiology.

## References

[B1] Nishiyori M Ueda H Prolonged gabapentin analgesia in an experimental mouse model of fibromyalgia Mol Pain 2008 4 52 10.1186/1744-8069-4-52 18990235 PMC2596100

[B2] Nishiyori M Nagai J Nakazawa T Ueda H Absence of morphine analgesia and its underlying descending serotonergic activation in an experimental mouse model of fibromyalgia Neurosci Lett 2010 472 184 187 10.1016/j.neulet.2010.01.080 20138970

